# Adaptive divergence and genetic vulnerability of relict species under climate change: a case study of *Pterocarya macroptera*

**DOI:** 10.1093/aob/mcad083

**Published:** 2023-07-06

**Authors:** Tian-Rui Wang, Hong-Hu Meng, Nian Wang, Si-Si Zheng, Yun Jiang, Duo-Qing Lin, Yi-Gang Song, Gregor Kozlowski

**Affiliations:** Eastern China Conservation Centre for Wild Endangered Plant Resources, Shanghai Chenshan Botanical Garden, Shanghai, 201602, China; Plant Phylogenetics and Conservation Group, Center for Integrative Conservation, Xishuangbanna Tropical Botanical Garden, Chinese Academy of Sciences, Kunming, 650223, China; State Forestry and Grassland Administration Key Laboratory of Silviculture in Downstream Areas of the Yellow River, College of Forestry, Shandong Agricultural University, Tai’an, 271018, China; Eastern China Conservation Centre for Wild Endangered Plant Resources, Shanghai Chenshan Botanical Garden, Shanghai, 201602, China; Eastern China Conservation Centre for Wild Endangered Plant Resources, Shanghai Chenshan Botanical Garden, Shanghai, 201602, China; Eastern China Conservation Centre for Wild Endangered Plant Resources, Shanghai Chenshan Botanical Garden, Shanghai, 201602, China; Eastern China Conservation Centre for Wild Endangered Plant Resources, Shanghai Chenshan Botanical Garden, Shanghai, 201602, China; Department of Biology and Botanic Garden, University of Fribourg, Fribourg, CH-1700, Switzerland; Eastern China Conservation Centre for Wild Endangered Plant Resources, Shanghai Chenshan Botanical Garden, Shanghai, 201602, China; Department of Biology and Botanic Garden, University of Fribourg, Fribourg, CH-1700, Switzerland; Natural History Museum Fribourg, Fribourg, CH-1700, Switzerland

**Keywords:** Climate change, genetic offset, genotype–environment association, RAD-seq, relict species

## Abstract

**Background and Aims:**

Understanding adaptive genetic variation and whether it can keep pace with predicted future climate change is critical in assessing the genetic vulnerability of species and developing conservation management strategies. The lack of information on adaptive genetic variation in relict species carrying abundant genetic resources hinders the assessment of genetic vulnerability. Using a landscape genomics approach, this study aimed to determine how adaptive genetic variation shapes population divergence and to predict the adaptive potential of *Pterocarya macroptera* (a vulnerable relict species in China) under future climate scenarios.

**Methods:**

We applied restriction site-associated DNA sequencing (RAD-seq) to obtain 8244 single-nucleotide polymorphisms (SNPs) from 160 individuals across 28 populations. We examined the pattern of genetic diversity and divergence, and then identified outliers by genetic differentiation (*F*_ST_) and genotype–environment association (GEA) methods. We further dissected the effect of geographical/environmental gradients on genetic variation. Finally, we predicted genetic vulnerability and adaptive risk under future climate scenarios.

**Key Results:**

We identified three genetic lineages within *P. macroptera*: the Qinling-Daba-Tianmu Mountains (QDT), Western Sichuan (WS) and Northwest Yunnan (NWY) lineages, which showed significant signals of isolation by distance (IBD) and isolation by environment (IBE). IBD and IBE explained 3.7–5.7 and 8.6–12.8 % of the genetic structure, respectively. The identified GEA SNP-related genes were involved in chemical defence and gene regulation and may exhibit higher genetic variation to adapt to the environment. Gradient forest analysis revealed that the genetic variation was mainly shaped by temperature-related variables, indicating its adaptation to local thermal environments. A limited adaptive potential was suggested by the high levels of genetic vulnerability in marginal populations.

**Conclusions:**

Environmental gradient mainly shaped the population differentiation of *P. macroptera*. Marginal populations may be at high risk of extinction, and thus proactive management measures, such as assisted gene flow, are required to ensure the survival of these populations.

## INTRODUCTION

Climate change potentially alters habitat suitability at the regional scale and results in local extinctions (Parmesan, 2006; Wiens, 2016), and has been considered to be a major driver of species range shifts and biodiversity loss (Scheffers *et al*., 2016; Waldvogel *et al*., 2020; Meng *et al*., 2021). Species may survive during climate change by tracking conditions to which they are currently adapted, exhibiting considerable phenotypic plasticity, and evolving adaptation strategies to new environments (Davis and Shaw, 2001; Parmesan, 2006; Capblancq *et al*., 2020; Waldvogel *et al*., 2020). For tree species with a long generation time, local adaptation is the most significant strategy to cope with climate change (Dauphin *et al*., 2020; Gougherty *et al*., 2021; Meng *et al.*, 2022). Thus, understanding and quantifying the adaptive potential of tree species could not only reveal how they would survive in the context of climate change, but also benefit conservation and management strategies to cope with global biodiversity loss (Razgour *et al*., 2019; Waldvogel *et al*., 2020).

Advances in landscape genomics have enabled the elucidation of the molecular genetic basis of the local adaptation of tree species (Li *et al*., 2017; Sork, 2018). Genotype–environment association (GEA) approaches can identify loci involved in local adaptation. The Mantel test and redundancy analysis (RDA) can detect the role of geographical or environmental variables in shaping genetic structure (Rellstab *et al.*, 2015). Genetic offset, which is measured by the locally adaptive allelic variation, can assess the amount of change in genomic composition required for a population to track future environmental conditions (Ellis *et al*., 2012; Capblancq *et al*., 2020). Therefore, landscape genomics offers powerful tools to detect adaptive genetic variation and predict the genetic vulnerability of species to climate change (Wang *et al*., 2021; Feng and Du, 2022).

Trees are the main components of forest ecosystems (Brodribb *et al*., 2020; Fazan *et al*., 2020). Tree species usually occupy heterogeneous environments, resulting in local adaptation (Savolainen, 2011; Sork *et al*., 2013; Capblancq *et al*., 2020). Assessing the impacts of environmental factors on local adaptation for tree species could help forecast the health of forest ecosystems (Sork *et al*., 2013; Sork, 2018). Relict species carry abundant genetic information related to environmental changes and function as storehouses of biodiversity (Woolbright *et al.*, 2014). For instance, relict species distributed in the Sino-Japanese and Sino-Himalayan Floristic Regions of East Asia exhibit high genetic diversity and contain valuable genetic resources (Qiu *et al*., 2011, 2017; Tang *et al*., 2018). However, these species are vulnerable to future climate changes due to genetic drift and limited gene exchange (Yannic *et al.*, 2014; Bay *et al.*, 2018; Cao *et al.*, 2020). At present, conservation management of relict species is mainly conducted from the perspective of genetic diversity (Chen *et al*., 2015; Wei *et al*., 2016; Wu *et al*., 2020; Xu *et al*., 2021). However, assessment of the genetic vulnerability and adaptation of relict species to future climate changes is more efficient and critical for the conservation and reforestation of these valuable resources (Capblancq *et al*., 2020; Gougherty *et al*., 2021). The rapid development of landscape genomics during the last decade has provided unprecedented opportunity to explore the genetic vulnerability of relict species.

*Pterocarya macroptera* is a vulnerable Cenozoic relict tree species in China that grows at an altitude of between 1100 and 3500 m (Lu *et al*., 1999; Song *et al*., 2019, 2020). It is a member of the family Juglandaceae and includes three varieties: var. *macroptera*, var. *insignis*, and var. *delavayi* (Lu *et al*., 1999). This species spans from 98°E to 120°E and exhibits considerable morphological variations (Song *et al*., 2020). With such a wide geographical range, whether environmental or geographical gradients shape the population differentiation of *P. macroptera*, to what extent population differentiation is influenced by environment or geography, and how populations would respond to future climate conditions remain poorly understood.

In this study, we sequenced 160 individuals of *P. macroptera* from 28 populations covering its entire distribution. Based on single-nucleotide polymorphisms (SNPs) obtained from restriction site-associated DNA sequencing (RAD-seq) data, we formulated the following objectives: (1) to infer population genetic differentiation and genetic diversity; (2) to quantify the contributions of environmental and geographical variables in shaping the spatial distributions of genetic variation; and (3) to assess the vulnerable populations with a mismatch between genotype and environment.

## MATERIALS AND METHODS

### Field sampling, RAD-seq library preparation and sequencing

Samples of healthy and mature leaves were collected from 28* P. macroptera* populations representing its entire geographical range. Samples were dried and kept in silica gel. Genomic DNA was extracted from tissue using a Plant Genomic DNA Kit (Tiangen, Beijing, China). A total of 160 individuals were selected for sequencing, with four to eight individuals per population (Supplementary Data Table S1). Samples were individually barcoded, and RAD libraries were prepared according to Baird *et al*. (2008). Genomic DNA was digested with the restriction enzyme *Taq*αI in a 30-μL reaction. Then adapter (containing individual unique 4- to 10-bp barcodes) ligation was performed on 200 ng DNA. Ligated DNA was pooled, purified, and PCR-amplified by an ABI GeneAmp 9700. DNA fragments with sizes between 300 and 500 bp were selected based on AMPure XP bead-based size selection steps. RAD libraries were sequenced on an Illumina HiSeq™ platform using 150-bp paired-end reads at Major Bio Pharm Technology, Shanghai, China.

### Data processing

The quality of the RAD data was assessed using FastQC (Andrews, 2010). Adapter sequences and low-quality bases (QC < 20) from the tail of each read were removed using Trimmomatic v0.36 (Bolger *et al*., 2014). Then, reads with length ~30 bp were discarded. The data quality was evaluated by calculating the GC content and the Q30 values. The cleaned paired reads were demultiplexed and trimmed to 120 bp in length using a next-generation sequencing-uniform script provided by Major Bio Pharm Technology. Variants were reference-aligned from the short reads using the STACKS v2.6.1 pipeline (Catchen *et al*., 2013; Davey *et al*., 2013). Firstly, the reference genome (*Pterocarya stenoptera*; Zhang *et al*., 2022) was indexed using BWA v0.7.17, and the paired-end reads of each sample were mapped to the reference genome based on the BWA-MEM algorithm (Li and Durbin, 2009). Next, the aligned reads were sorted by SAMTOOLS v1.15.1 (Li *et al*., 2009), and the gstacks module in STACKS was used to create loci using a sliding window algorithm with default parameters. Finally, using the populations module in STACKS, the SNPs were obtained and filtered according to the following criteria: (1) markers with an observed heterozygosity > 0.70 among individuals were removed to reduce the potential occurrence of paralogues; (2) minor allele frequency (MAF) < 0.05; (3) only the first SNP locus of each read was retained by parameter write_single_snp to reduce physical linkage; and (4) a minimum of 70 % of individuals within a population were required to process a locus.

VCFtools v0.1.13 was used to discard loci with missing data present in at least 20 % of individuals and to keep only biallelic SNPs (Danecek *et al*., 2011). Variant sites described in the VCF format were functionally annotated based on the *P. stenoptera* gene models using SnpEff v4.3t (Cingolani *et al*., 2012). For SNPs, we performed an online BLAST search via the National Center for Biotechnology Information (NCBI) website based on the protein sequences of genes annotated by SnpEff. Based on the highest percentage identity of BLAST alignment, combined with the results of each gene identified by the UniProt database (https://www.uniprot.org/), we determined the possible molecular function of each gene and the biological process involved.

### Population structure and genetic diversity

We investigated the maximum likelihood ancestor of each individual for all SNPs (obtained above) using ADMIXTURE v1.30 (Alexander *et al*., 2009; Alexander and Lange, 2011). For ADMIXTURE, the range of the co-ancestry clusters (*K*) was set to vary between 1 and 10. The optimal number of clusters was determined based on the lowest cross-validation error rate. For each *K* value, we performed a 10-fold cross-validation. The genetic differentiation among populations was calculated based on all SNPs using principal component analysis (PCA) in the R package adegenet 2.1-5 (Jombart *et al*., 2010; R Core Team, 2019). Population differentiation (*F*_ST_) among lineages was calculated based on different SNP datasets using VCFtools v0.1.13 (Danecek *et al*., 2011). We calculated the number of private alleles (*N*_P_), percentage of polymorphic loci (PL), mean observed heterozygosity (*H*_O_) and mean expected heterozygosity (*H*_E_) using PLINK v1.9 with default settings based on all SNPs and all outlier SNPs (Purcell *et al*., 2007). To minimize differences resulting from sample size variations, we also calculated unbiased allele richness for all SNPs and all outlier SNPs using the R package hierfstat 0.5-11 (Goudet, 2005; Halbert and Derr, 2008). The unbiased estimation of nucleotide diversity (*π*) was conducted using pixy v1.2.7 for all loci (including non-polymorphic loci) and all outlier loci following a window length of 10 kb (Korunes and Samuk, 2021). For each summary statistic, Tukey HSD tests were used to evaluate significant differences.

### Environmental variables and their responses to genetic variation

Nineteen bioclimatic variables for the current period (1970–2000) at 30 arcsec resolution were downloaded from the WorldClim v.2.1 database based on the geographical coordinates of the sampled populations (Fick and Hijmans, 2017). Elevation was also included as it may be important to alpine species. We further downloaded predicted environmental variables in the defined period (2081–2100) using low- and high-emission scenarios of the shared socioeconomic pathway (SSP126 and SSP585; Meinshausen *et al.*, 2020). Gradient forest (GF) analysis was performed to identify 20 environmental variables that best explained the distribution of genetic variation using the R package gradientForest 0.1-17 based on all SNPs (Ellis *et al*., 2012). Gradient forest models apply a non-parametric machine-learning regression algorithm tree to explore non-linear associations of spatial, environmental and allelic variables (Fitzpatrick and Keller, 2015). Two thousand regression trees were used for each SNP to fit the GF model, while keeping all parameters as default values. After evaluating the ranked accuracy importance among 20 environmental variables, the absolute value of Pearson correlation coefficients among these environmental variables was evaluated using the R package usdm 1.1-18 (Naimi *et al*., 2014). Environmental variables across the range of *P. macroptera* with correlation coefficients |*r*| < 0.7 and the top three variables identified by the GF analysis were retained. Finally, eight variables (BIO02, mean diurnal range; BIO03, isothermality; BIO04, temperature seasonality; BIO07, temperature annual range; BIO08, mean temperature of wettest quarter; BIO13, precipitation of wettest month; BIO15, precipitation seasonality; and elevation) were used for subsequent analysis (Supplementary Data Tables S2 and S3). The eight environmental variables were used to build the final GF model, which was used to predict the genetic vulnerability of each population across the range of *P. macroptera* (see section Prediction of genetic vulnerability). Given that the correlation between the top three variables identified by GF and the other variables may affect the final prediction of GF, we also used bioclimatic variables with correlation coefficients |*r*| < 0.7 (i.e. BIO02, BIO07, BIO08, BIO13 and BIO15) to build the final GF model.

### Identification of selection signatures

The *F*_ST_-based methods identified outlier SNPs as those with levels of differentiation above those of neutral SNPs among populations (Beaumont and Nichols, 1996). The GEA-based methods identified outlier SNPs as those exhibiting allele frequency changes that were strongly related to environmental variables (De Mita *et al*., 2013). We focused on the *F*_ST_-based (Whitlock and Lotterhos, 2015; Luu *et al*., 2017) and GEA-based approaches (Günther and Coop, 2013; Caye *et al*., 2019) to detect outlier SNPs that were subjected to selection.

The R packages PCADAPT 4.3.3 and OutFLANK 0.2 were employed as *F*_ST_-based methods to identify *F*_ST_ outlier SNPs. These two methods were shown to have the lowest false discovery rate (FDR) (Luu *et al*., 2017). PCADAPT identifies outlier SNPs with respect to population structure through PCA (Privé *et al*., 2020). Three principal components (*K* = 3) captured most background genetic variation based on the results of ADMIXTURE and PCA (see section Genomic divergence and genetic diversity). The SNPs that deviated significantly from the neutral background structure along the principal components (Bonferroni correction with adjusted *P*-values < 0.05) were identified as putatively genetic differentiation loci. OutFLANK employs an improved likelihood approach to estimate the null distribution of population differentiation for neutral loci (Whitlock and Lotterhos, 2015). This program does not take demographic history into account and is less prone to false positives (Whitlock and Lotterhos, 2015). We used a left and right trim value of 0.05 for the null distribution of *F*_ST_ with default settings. SNPs with a minimum heterozygosity for loci > 0.1 and the desired FDR threshold < 0.05 were identified as putatively genetic differentiation loci.

Bayesian mixed modelling and mixed linear modelling were implemented in BAYENV (Coop *et al*., 2010; Günther and Coop, 2013) combined with latent factor mixed modelling (LFMM; Caye *et al*., 2019) to investigate the genomic basis of adaptation across contemporary climate gradients. A null hypothesis empirical model was established using putatively non-*F*_ST_ genetic loci (removed loci identified by PCADAPT and OutFLANK) in BAYENV. An allele frequency versus environment variable covariance matrix among populations was calculated with 10^6^ iterations in five independent runs. Based on the prior null hypothesis distribution model, we calculated the posterior distribution *P-*values of the correlation between allele frequency and environmental variables. The Bayes factor (BF) was generated by calculating the correlation between the allele frequency and environmental variable covariance matrices after 10^5^ runs. The BF was calculated five times, and the average BF was treated as the final matrix of the BF. SNPs with BF values > 10 and among the top 5 % with absolute value of Spearman rank correlation coefficients (*ρ*) were considered as significantly environment-associated loci. To reduce the FDR, the associations between genotypes and environmental variables were determined using the LFMM method, which considers the population structure. Genetic data were converted into LFMM format using the R package LEA 2.0.0 (Frichot and François, 2015). Based on the clustering analysis results, *K* = 3 was selected as the optimal number of latent factors for sparse non-negative matrix factorization (SNMF). Missing SNP data were imputed based on the inferred genetic structure using the built-in SNMF function in the LEA package. Then, we ran ten independent operations to simulate the correlation between allele frequency and environmental variables for a burn-in period of 5,000 steps followed by 10,000 iterations. We used the lfmm.pvalues function in the LEA package to adjust the *P*-values. SNPs with adjusted *P*-values < 0.001 strongly support associations between allele frequencies and environmental variables. To verify the robust detection of outlier loci, we further corrected the *P*-values at an FDR of 0.05 using the R package fdrtool 1.2.17 (Strimmer, 2008).

Using the results of these four approaches, we divided our dataset into four categories for the subsequent analyses: (1) all SNPs; (2) putative *F*_ST_ SNPs detected jointly by PCADAPT and OutFLANK; (3) putative GEA SNPs detected jointly by BAYENV and LFMM; and (4) putative outlier SNPs identified by the *F*_ST_ and GEA methods. Finally, we used a Venn diagram to evaluate the consistency of outlier SNPs identified across the *F*_ST_ and GEA methods, respectively.

### Inferring isolation by distance and isolation by environment

Isolation by distance (IBD) and isolation by environment (IBE) were inferred using the R package vegan 2.5.6 to investigate the role of geographical and environmental variables in shaping spatial genetic differentiation (Forester *et al*., 2018; Oksanen *et al*., 2019). The geographical distance (pairwise Euclidean difference, representing IBD) between sites was calculated using the R package geosphere 1.5.14 (Hijmans *et al.*, 2021). The eight environmental variables were first scaled and centralized to account for differences in magnitude, and then were used to calculate the environmental distance (Bray–Curtis distance, representing IBE) using the R package ecodist 2.0.9 (Goslee and Urban, 2007). The genetic distance (pairwise *F*_ST_/(1 − *F*_ST_)) was calculated based on all SNPs using the R package hierfstat 0.5.11 (Goudet, 2005). Simple Mantel tests (Mantel, 1967; Diniz-Filho *et al*., 2013) were used to test whether genetic distance was significantly correlated with geographical or environmental distance. Partial Mantel tests were used to investigate the independent effects of geographical/environmental distance on genetic distance. We further investigated the correlation between environmental and geographical distance. The level of significance of the Mantel tests was assessed with 999 permutations.

### Redundancy analysis

We used RDA to assess the relative contribution of environmental and geographical distances to population genetic differentiation using the R package vegan 2.5-6 (Forester *et al*., 2018; Oksanen *et al*., 2019). Allele frequency matrices for each population, as well as two independent matrices of environmental and geographical variables, were used for RDA tests. Considering that the RDA test does not allow for the presence of missing allele frequency data, we used the R package LEA 2.0.0 (Frichot and François, 2015) to impute missing allele frequencies for each population. Geographical variables were defined by geographical distances using principal coordinates of neighbourhood matrices (PCNMs) in the R package vegan (Oksanen *et al*., 2019). The first half of the PCNM variables (PCNM1, PCNM2, PCNM3 and PCNM4) with significant positive eigenvalues were retained, as has been suggested by Fitzpatrick and Keller (2015). Following the recommendation of Borcard *et al*. (2011), we used all SNPs to perform forward selection for both geographical and environmental variables with an α value of 0.05 to avoid overfitting. This resulted in the retention of three PCNMs (PCNM1, PCNM2 and PCNM3) and seven environmental variables (BIO02, BIO03, BIO04, BIO07, BIO13, BIO15 and elevation) for the subsequent analyses. Full and partial RDA model tests for different SNP sets (all and *F*_ST_, GEA and all outlier SNPs) were performed to distinguish the independent effects of environment and geography by reciprocally constraining geographical and environmental variables. Significance was assessed using the randomization procedure implemented in the function ANOVA.cca with 999 randomizations.

### Prediction of genetic vulnerability

To predict genetic vulnerability under future climate conditions, we performed GF (Ellis *et al*., 2012; Fitzpatrick and Keller, 2015) and risk of non-adaptedness analysis (RONA; Rellstab *et al*., 2016; Pina-Martins *et al*., 2018) using all SNPs and GEA SNPs. Gradient forest analysis was used to identify the spatial regions in which genotype–environment relationships are most likely to be disrupted by climate change. We evaluated the mismatch between current and predicted genomic compositions under future environmental projections during 2081–2100 under low- and high-emission scenarios (SSP126 and SSP585). Eight environmental variables were included in the GF model to predict the genomic composition of each grid point across the range of *P. macroptera*. The GF model was tested using 2000 regression trees per SNP. The Euclidian distance between current and future genetic compositions was calculated; this represents the scale of genetic change needed to match environmental change (i.e. genetic offset), with higher values indicating greater vulnerability of the population (Fitzpatrick and Keller, 2015). To illustrate the regions predicted to experience greater impacts under future environments with a lack of adaptive evolution or migration (Fitzpatrick and Keller, 2015), the genetic offset was visualized as landscape maps for all SNPs and GEA SNPs.

We used RONA to quantify the theoretical average change in allele frequency under predicted future climate scenarios and then predicted the adaptive potential of species under these scenarios. First, the allele frequency of each individual was obtained using the R package LEA 2.0.0 (Frichot and François, 2015). A regression model was constructed based on the allele frequencies and eight environmental variables. The theoretically expected allele frequencies during 2081–2100 under the SSP126 and SSP585 emission scenarios were then predicted based on the regression model. The average difference between the current and predicted allele frequencies was the RONA value, which represented the adaptive potential of the population under future climate conditions. A higher RONA value indicates a lower potential (high genetic vulnerability) of the population to adapt to future climate conditions. We calculated the weighted mean *R*^2^ of the regression model for each population, as recommended by Pina-Martins *et al*. (2018). Finally, we identified the top three environmental variables that were most tightly associated with all SNP and GEA SNP sets.

## RESULTS

### Genomic data

A total of 1 125 776 716 clean paired-end reads were obtained from 160 individuals of *P. macroptera*. An average of 7 036 104 reads was retained per individual. The average mapping rate was ~77 % (range 60–94 %; Supplementary Data Tables S1 and S4). We retained 8244 high-quality SNPs after stringent quality control (Supplementary Data Table S5). The annotation of the 8244 SNPs is provided in Supplementary Data Table S6. Of the 8244 SNPs, 1779 (21.6 %) resided in coding regions. The remaining SNPs (6465 SNPs, 78.4 %) resided in upstream gene variants (1812 SNPs), downstream gene variants (1257 SNPs), intron variants (859 SNPs) and intergenic regions (2537 SNPs; Supplementary Data Table S6).

### Genomic divergence and genetic diversity

**Fig. 1.**
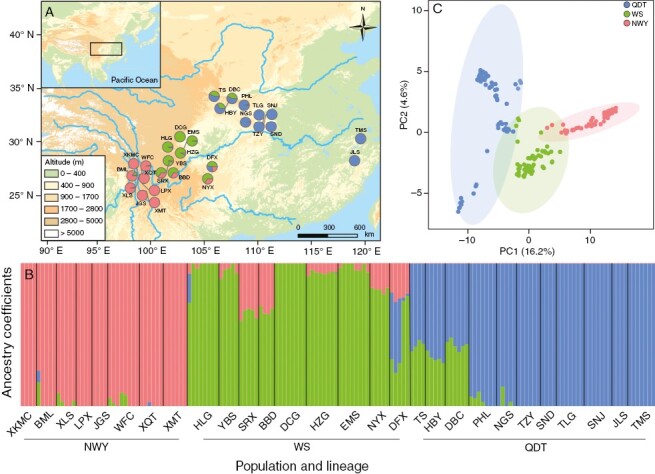
Geographical distribution and population genomic structure in *P. macroptera*. (A) Mapping of the Bayesian genetic clusters inferred by ADMIXTURE. Colours represent different ancestry groups. (B) Ancestry assignment for 160 individuals in 28 populations of *P. macroptera* at *K* = 3. Each bar represents an individual, with different colours reflecting varying ancestry. (C) PCA with different colours reflecting different groups.

Based on the 8244 SNPs, ADMIXTURE identified *K* = 3 as the most likely number of evolutionary clusters among the 28 populations (Fig. 1A, B; Supplementary Data Fig. S1). We detected three distinct clusters: the Qinling-Daba-Tianmu Mountain (QDT), Western Sichuan (WS) and Northwest Yunnan (NWY) lineages. PCA yielded a similar grouping, with the first two PCs accounting for 20.8 % of the total genetic variation using 8244 SNPs (16.2 and 4.6 % for PC1 and PC2 respectively; Fig. 1C). The genetic differentiation based on the different SNP datasets consistently supported the highest genetic differentiation between the QDT and NWY lineages, followed by the NWY and WS lineages, while the QDT and WS lineages had the lowest genetic differentiation (Table 1). In addition, four SNP datasets revealed different levels of genetic differentiation. Among them, the differentiation level based on *F*_ST_ SNPs was 0.32–0.66, which was higher than that based on GEA SNPs (0.29–0.61) and all outlier SNPs (0.25–0.58). Based on all SNPs, the genetic differentiation among different lineages was the lowest (0.09–0.28). For all SNPs, the genetic diversity indicated no significant difference among the three lineages (*P* < 0.05; Table 2; Supplementary Data Tables S7 and S8). For all outlier SNPs, the WS lineage showed more polymorphic loci and higher allelic richness (AR) than the NWY and QDT lineages. Specifically, the HLG population in the WS lineage had the greatest proportion of polymorphic loci (53.49 %) and the DFX population had the highest allelic richness (1.39) among the 28 populations (Supplementary Data Table S7).

**Table 1.** Genetic differentiation (F_ST_) on all 8244 SNPs and all outlier SNPs (in parentheses, below the diagonal) and on F_ST_ SNPs (PCADAPT and OutFLANK, 932 SNPs) and GEA SNPs (BAYENV and LFMM, 957 SNPs, in parentheses, above the diagonal) between groups of *P. macroptera*.

	NWY lineage	WS lineage	QDT lineage
NWY lineage	–	0.478 (0.382)	0.656 (0.609)
WS lineage	0.155 (0.383)	–	0.323 (0.288)
QDT lineage	0.281 (0.575)	0.084 (0.249)	–

**Table 2.** Genetic diversity for *P. macroptera* under all SNPs and all outlier SNPs.

Lineage	*N*	*N*_P_	PL	*H*_O_	*H*_E_	*π*	Allelic richness
All SNPs							
NWY	5.25^a^	0.00^a^	33.54^a^	0.11^a^	0.10^a^	0.12^a^	1.27^a^
WS	6.22^a^	0.33^a^	45.16^a^	0.12^a^	0.11^a^	0.13^a^	1.31^a^
QDT	5.63^a^	0.27^a^	37.08^a^	0.11^a^	0.10^a^	0.11^a^	1.27^a^
All outlier SNPs							
NWY	5.25^a^	0.00^a^	30.98^b^	0.09^a^	0.10^ab^	0.12^ab^	1.26^b^
WS	6.22^a^	0.11^a^	43.64^a^	0.12^a^	0.13^a^	0.15^a^	1.33^a^
QDT	5.63^a^	0.09^a^	30.00^b^	0.09^a^	0.09^b^	0.10^b^	1.24^b^

*N*, number of individuals; *N*_P_, number of private alleles; PL, percentage of polymorphic loci; *H*_O_, mean observed heterozygosity; *H*_E_, mean expected heterozygosity; *π,* mean nucleotide diversity.

Indices with different superscript letters represent significant differences (*P* < 0.05, Tukey–HSD).

### Putative outlier SNP detection

A total of 932 outlier SNPs were detected using the *F*_ST_-based methods, while 537 and 697 outlier SNPs were identified by PCADAPT and OutFLANK, respectively (Table 3; Supplementary Data Table S9; Supplementary Data Fig. S2). The number of top 20 % outlier SNPs identified by PCADAPT was 117, and that by OutFLANK was 139, with 17 outlier SNPs overlapping between the two methods (Supplementary Data Fig. S3; Supplementary Data Table S10). Among these 17 overlapping outlier SNPs, ten were located in ten genes and the remaining seven SNPs were located in intergenic regions. Of the ten genes, the PST000227 gene was not identified by the NCBI BLAST analysis and the PST000586 gene had no known function. Three genes (PST026568, PST019941 and PST000475) were involved in embryo development ending in seed dormancy, the triterpenoid biosynthetic process and the reproductive development process (Supplementary Data Table S10).

**Table 3.** Number of candidate SNP loci under putative selection identified by PCADAPT, OutFLANK, BAYENV and LFMM.

Method		SNPs	BIO02	BIO03	BIO04	BIO07	BIO08	BIO13	BIO15	Elevation
*F*_ST_-based	PCADAPT	537								
	OutFLANK	697								
	*F*_ST_ total	932								
GEA-based	BAYENV	431	80	207	166	183	8	28	124	20
	LFMM	796	102	167	186	201	132	353	222	149
	GEA Total	957	162	288	290	306	133	356	283	157
	All outlier SNPs^a^	1380								

^a^SNPs identified by *F*_ST_- and GEA-based methods.

Based on the GEA approach, a total of 957 outlier SNPs putatively associated with environmental variables were detected. Among them, 431 and 796 SNPs were detected by BAYENV and LFMM (FDR < 0.05), respectively (Table 3; Supplementary Data Table S11; Supplementary Data Figs S4 and S5). We found 356, 306, 290, 288 and 283 SNPs associated with the environmental variables BIO13, BIO07, BIO04, BIO03 and BIO15, respectively. BAYENV and LFMM detected 49 and 47 SNPs simultaneously associated with the top four environmental variables (BIO04, BIO03, BIO07, and BIO15; see next section) (Supplementary Data Fig. S6A, B). Eight SNPs were detected by both BAYENV and LFMM (Supplementary Data Fig. S6C). Among these eight SNPs, one was located in an intergenic region, and the remaining seven SNPs were located in seven genes, respectively. Of the seven genes, the PST000586 and PST036282 genes had no known function. The PST020689 and PST029083 genes were involved in the regulation of gene expression by stress factors and photosynthesis, respectively. In addition, the PST029941 gene was involved in the glycolipid and sulfolipid biosynthetic process, and the PST018122 gene was responsible for the diterpenoid, sesquiterpene and terpenoid biosynthetic process (Supplementary Data Table S10). A total of 16 genes were detected by the *F*_ST_ and GEA approaches, among which the PST035198 gene, involved in the regulation of gene expression, was detected by both methods.

### Environmental and spatial associations with genetic variation

**Fig. 2.**
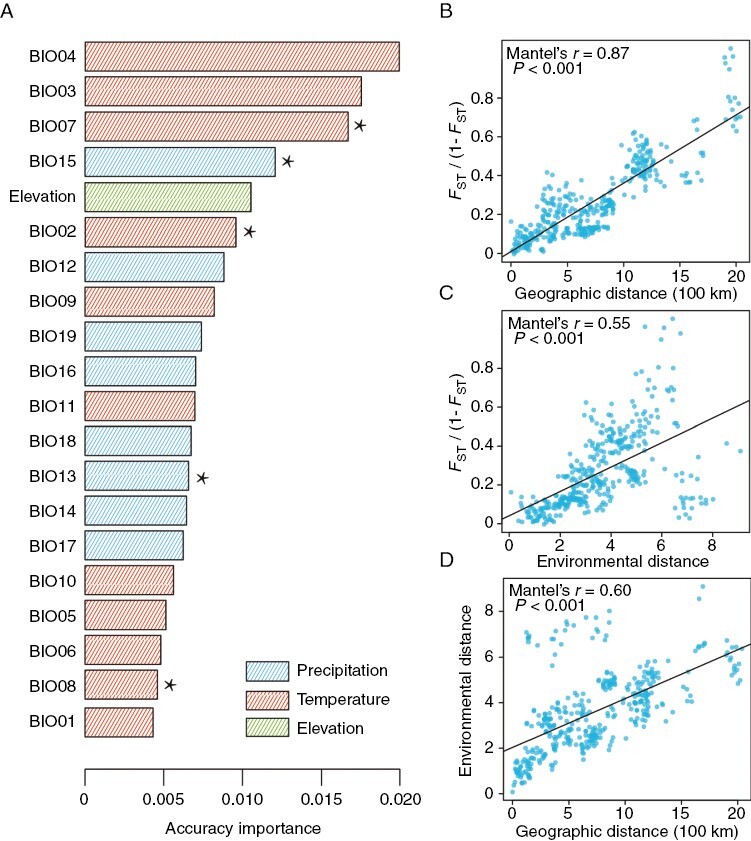
(A) Ranked importance of environmental variables based on GF modelling. The asterisks represent the uncorrelated environment variables (Pearson's |*r*| < 0.7). (B–D) Mantel tests of genetic distance [*F*_ST_/(1 − *F*_ST_)] against (B) geographical distance and (C) environmental distance, and (D) correlation between geographical distance and environmental distance.

The GF analysis revealed significant differences in genetic composition along the geographical range of *P. macroptera* (Fig. 2A; Supplementary Data Table S12). Temperature seasonality (BIO04) was identified as the most important predictor among the environmental variables considered, followed by BIO03, BIO07, BIO15 and elevation. In addition, allelic composition changed sharply along the temperature-related top three environmental variables, BIO04, BIO03, and BIO07 (Supplementary Data Fig. S7).

Using all SNPs, the pairwise genetic distance between locations [*F*_ST_/(1 − *F*_ST_)] was highly correlated with geographical distance, suggesting a strong signal of IBD (Fig. 2B). Genetic distance was also significantly associated with environmental distance based on eight environmental variables (Fig. 2C). In a partial Mantel test, only geographical distance was significant (Supplementary Data Table S13), suggesting that this is the strongest force structuring genome-wide variation. In addition, the autocorrelation between environmental and geographical distances was also strong (Fig. 2D).

**Fig. 3.**
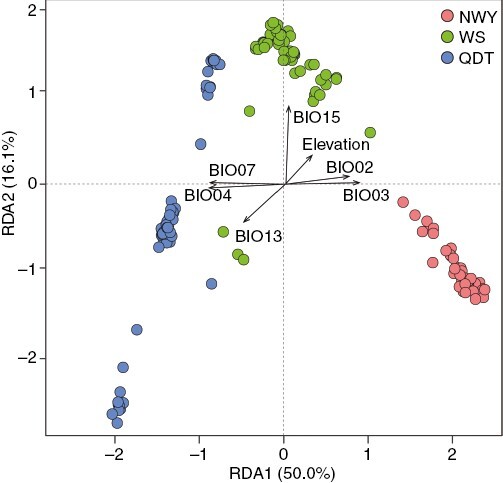
Partial RDA partitioning sources of genetic differentiation among populations in *P. macroptera* into environment by condition geography using all outlier SNPs. The plot shows the first and second RDA axes with individuals as coloured circles and environmental variables as black vectors.

Isolation by distance explained 3.7–5.7 % of the variation after controlling for environment, while IBE explained 8.6–12.8 % of the variation after controlling for geography (Table 4; Supplementary Data Table S14). Based on the four SNP datasets, the contribution of environment to genetic variation was higher than that of geography. Thus, we further identified the explanatory environmental variables for the genetic variation in different lineages of *P. macroptera* using different SNP sets. Considering the similar results of RDA and partial RDA, we reported RDA results with a higher proportion of explained genetic variation. Based on the four SNP datasets, the contribution of environmental variables to genetic variation in the three lineages was generally consistent (Fig. 3; Supplementary Data Fig. S8). Precipitation-related variables (BIO15, BIO13) and elevation contributed most to the genetic variation in the WS lineage. Temperature-related variables (BIO04, BIO07) explained most of the genetic variation in the QDT lineage. Mean diurnal range (BIO02) and isothermality (BIO03) contributed most to the genetic variation in the NWY lineage. A total of 28.6–60.1 % of the variation was explained by two components (environment and geography) of the different SNP sets (‘Total explained’ in Table 4), and a large proportion of total genetic variation was explained by their combined effect (‘Total confounded’ in Table 4). This combined effect was most pronounced in the *F*_ST_ SNPs.

**Table 4.** *Summary of genetic differentiation associated with environmental, geographical and their combined effects based on redundancy analysis in *P. macroptera**.

	All SNPs 8244	*F*_ST_ SNPs 932	GEA SNPs 957	All outlier SNPs 1380
Combined fractions				
F~geog.	0.200***	0.481***	0.448***	0.415***
F~env.	0.249***	0.545***	0.533***	0.484***
Individual fractions				
F~geog. | env.	0.037***	0.057***	0.042***	0.050***
F~env. | geog.	0.086***	0.121***	0.128***	0.119***
Total explained	0.286***	0.601***	0.576***	0.534***
Total confounded	0.163	0.423	0.406	0.365
Total unexplained	0.714	0.399	0.424	0.466
Total	1.000	1.000	1.000	1.000

F, dependent matrix of minor allele frequencies; RDA tests are of the form: F ~ independent matrices | covariate matrices. env., seven retained environmental variables; geog., three retained principal coordinates of neighbourhood matrices variables. Total explained, total adjusted *R*^2^ of individual fractions; Total confounded, total of individual fractions confounded between various combinations of environment and geography.

***P ≤ 0.001.

### Landscape of future predictions

**Fig. 4.**
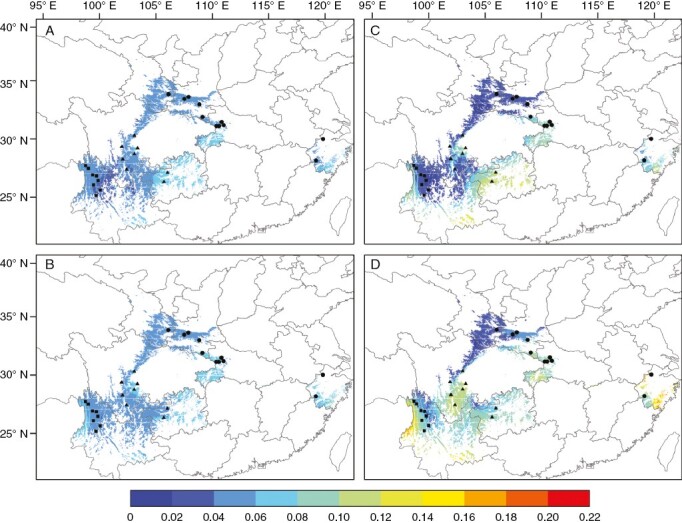
Prediction of genetic offset to future climate change based on eight environment variables for (A, B) all SNPs and (C, D) GEA SNPs. (A) and (C) reflect scenario SSP126 2081–2100; (B) and (D) reflect scenario SSP585 2081–2100. Colour scale at bottom represents genomic offset. Black dots represent sampling sites on the geographical map, with squares, triangles and circles representing the Northwest Yunnan (NWY), Western Sichuan (WS) and Qinling-Daba-Tianmu Mountain (QDT) lineage, respectively.

Gradient forest prediction based on five environmental variables was similar to those based on the eight environment variables (Supplementary Data Fig. S9). Given the similarity of the results, we reported predictions based on the eight environment variables. The ranges and degree of genetic mismatch increased under SSP585 compared with those under SSP126 based on all SNPs and GEA SNPs. Comparing the two scenarios, we calculated the proportion of the distribution range having a genetic mismatch > 50 % of the maximum detected value (0.22 in this study; Fig. 4). There was no distribution space exceeding the threshold of vulnerability based on all SNPs. Based on GEA SNPs, 3.4 and 6.9 % of the distribution space was recognized as above the threshold of vulnerability under SSP126 and SSP585, respectively. The eastern and western peripheral populations of *P. macroptera* were most vulnerable under both climate scenarios. This indicates that these populations may be confronted with climate-induced selective pressure in the future. The northern populations of the QDT lineage and the eastern populations of the NWY lineage had low genetic vulnerability (Fig. 4).

**Fig. 5.**
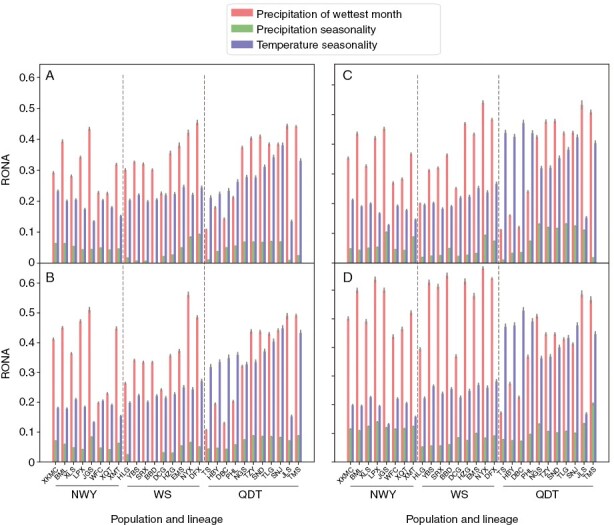
RONA plot for the three most associated environmental variables for (A, B) all SNPs and (C, D) GEA SNPs. (A) and (C) reflect scenario SSP126 2081–2100; (B) and (D) reflect scenario SSP585 2081–2100. Bars represent the weighted means (by *R*^2^ value) and lines at the tops of bars represent the standard error.

The RONA suggested that GEA SNPs had a higher genetic vulnerability than all SNPs (Fig. 5; Supplementary Data Table S15). Most populations under the high-emission scenario had a higher genetic vulnerability than those under the low-emission scenario. Precipitation of wettest month (BIO13), temperature seasonality (BIO04) and precipitation seasonality (BIO15) played a primary role in vulnerability. The southernmost and easternmost marginal populations of *P. macroptera*, such as the populations JGS, DFX, NYX, TMS and JLS, had a lower adaption potential for BIO13. Populations isolated at the southern and eastern edge of their natural distribution area may face higher vulnerability under predicted future climate scenarios.

## DISCUSSION

### Genomic divergence and diversity

We found three genetic clusters within *P. macroptera* with some admixed individuals between them. Two genetic clusters (NWY and WS) were located in the Sino-Himalayan Forest subkingdom and one (QDT) in the Sino-Japanese Forest subkingdom. This pattern of population structure is consistent with previous studies in East Asia (Wu and Wu, 1996; Qiu *et al*., 2011, 2017; Chen *et al*., 2015; Ma *et al*., 2015; Cao *et al*., 2016, 2020; Wei *et al*., 2016). The two genetic clusters within the Sino-Himalayan Forest subkingdom seem to mirror the similar phylogeographic break in many other species (Meng *et al*., 2015; Yang *et al*., 2017; Luo *et al*., 2018; Wang *et al*., 2019; Nocchi *et al.*, 2023). The level of genetic differentiation among *P. macroptera* populations is higher than that in other closely related wingnut taxa (*P. stenoptera*: 0.067) (Li *et al*., 2018). The high genetic differentiation between the three lineages within *P. macroptera* may be related to the geographical isolation and divergent selection. Some SNPs located in different lineages may be subject to divergent selection in the homogenous gene pool. The WS lineage, mainly located in the west of the Sichuan Basin, has a higher level of genetic diversity, possibly due to the presence of an ancient refugium, which provided stable and suitable conditions for maintenance of genetic diversity (López-Pujol *et al*., 2011; Tang *et al*., 2018). Previous reports on plants (Li *et al*., 2023), birds (Wu *et al*., 2017), amphibians (Qiao *et al*., 2018) and insects (Tang *et al*., 2022) have shown that mountains around the Sichuan Basin harboured suitable microenvironments for species, especially for relict species. Another possible explanation for the high genetic diversity of the WS lineage could be the mixed populations in this lineage (i.e. SRX, BBD, BYX, DFX) receiving genetic introgression from the NWY and QDT lineages and thus providing higher genetic diversity for the WS lineage. The low genetic diversity in the QDT and NWY lineages requires further work to investigate the evolutionary dynamics of extinction, colonization and effective population size in these two lineages.

### Landscape of current adaptive genetic variation

Trees often occupy highly heterogeneous environments and ecologically adapt to the local environment (Capblancq *et al*., 2020). Hence, the signature of environmental isolation of *P. macroptera* likely reflects local environmental adaptation (Nachman and Payseur, 2012; Wang and Bradburd, 2014). Temperature regimes and precipitation patterns are critical factors that define species distribution and plant growth (Root *et al*., 2003; Wahid *et al*., 2007). Redundancy analysis showed that temperature-related factors were important in accounting for the adaptive variation of *P. macroptera*. This is similar to other findings that temperature also impacts the adaptive genetic variation of *P. stenoptera* (Li *et al*., 2018, 2022), a species closely related to *P. macroptera*. We are not sure if the same subsets of loci are involved in the local adaptation of both *P. macroptera* and *P. stenoptera*. Further research is needed to determine this. The top three environmental variables explaining genetic variation were temperature-related, especially for the NWY and QDT lineages. *Pterocarya* is a typical riparian relict tree genus (Song *et al*., 2020, 2021). Compared with other *Pterocarya* species, *P. macroptera* occupies the highest elevations, with the largest change in elevational range, along mountain streams and water-rich slopes (Kozlowski *et al*., 2018; Song *et al*., 2019). This is likely why the other two important factors that drove genetic variation in *P. macroptera* were precipitation seasonality and elevation, which were also the top two factors for the WS lineage. The uplift of the Qinghai–Tibet Plateau created elevation gradients in East Asia, and intensification of the East Asian monsoon system shaped temperature regimes and precipitation patterns (Qiu *et al*., 2011, 2017), which are the most suggestive factors responsible for genetic variation and the major phylogeographic breaks among the three lineages.

The GEA-based methods identified seven SNPs located in seven genes that were associated with environmental variables (BIO04, BIO03, BIO07, and BIO15). The identified GEA genes involved in chemical defence and gene regulation may exhibit high genetic variation to adapt to the environment (temperature and precipitation). The PST018122 and PST019941 genes (detected by the *F*_ST_ method) were both involved in the triterpenoid biosynthetic process. As significant chemical defence compounds for the growth and development of plants and for coping with a stressed environment, terpenoids directly act as antimicrobials or signals for resisting herbivores and other natural enemies (Dudareva *et al*., 2004; Paschold *et al*., 2006). Terpenoids dominate in the leaves of *Pterocarya* species (Xiao *et al*., 2002; Liu *et al*., 2004; Zhang *et al*., 2006; Ebrahimzadeh *et al*., 2009; Yin *et al*., 2019), suggesting that terpene biosynthesis-related genes may play an important role in protecting the leaves of *P. macroptera*. The PST020689 gene was involved in the regulation of gene expression by stress factors. Regulation of biological processes is responsible for stress resistance under biotic and abiotic stresses (Licausi *et al*., 2013). Genes related to stress resistance may be of significance to *P. macroptera* under stressed conditions. The regulatory expression of these identified genes may be the genomic imprint for the local adaptation of *P. macroptera* to its habitats.

### Genetic vulnerability under future climate conditions

Understanding the genetic basis of adaptation and determining the adaptive ability of species to future climate conditions are crucial in the context of climate change (Fitzpatrick and Keller, 2015; Dauphin *et al*., 2020). Previous studies reported relevant strategies to gain insight into the potential risk of species persistence under climate change (Bay *et al*., 2018; Du *et al*., 2020; Zhao *et al*., 2020; Vranken *et al*., 2021; Sang *et al*., 2022). We found that marginal populations of *P. macroptera* had a high level of genetic vulnerability, suggesting that these populations are potentially at higher risk of *in situ* extinction under future climate changes. These marginal populations may be less resilient to future climates because genotypes were not sufficiently correlated with predicted climate variables. This result reinforces our understanding that ecologically marginal populations may be separated not only by distance from the core of the species’ distribution but also experience different biotic and abiotic environments (Munwes *et al*., 2010; Santini *et al*., 2019). Although the static elevation variable used for the prediction could have led to an underestimation of genetic vulnerability, riparian forest niches limited the movement of populations to higher elevations. In addition, the strong geographical isolation limited the ability of populations to track spatial changes through migration.

The genetic vulnerability of *P. macroptera* predicted in this study may be related to the species-specific tolerance to environmental variables and the complex topography of mountains. Overall, we expect that long-term sustained climate change will result in marginal populations with high genetic vulnerability (Dauphin *et al*., 2020). These highly vulnerable populations need to adapt quickly to climate change. Otherwise they may be at risk of extinction (Franks and Hoffmann, 2012; Capblancq *et al*., 2020). Future work needs to integrate adaptive genetic variation with biogeographic models to accurately identify species vulnerability under future climate conditions.

### Implications for conservation management

There is a growing interest in evolutionarily informed management strategies that rely on the spatial distribution of genetic diversity and genetic vulnerability (Aitken and Whitlock, 2013; Lefèvre *et al*., 2014; Gougherty *et al*., 2021). Hence, a comprehensive understanding of the spatial genetic diversity helps to develop conservation strategies (Petit *et al*., 1998). Populations with high genetic diversity may have a greater potential in adaption to climate change and may harbour valuable breeding materials (Frankel *et al*., 1995). Therefore, priority should be given to conservation of such populations, such as populations of the WS lineage of *P. macroptera*.

Gradient forest analysis and RONA have been widely used in assessing genetic vulnerability. Our results suggested that marginal populations of *P. macroptera* have higher genetic vulnerability. Assisted gene flow from populations with genotypes preadapted to future climate may help those marginal populations mitigate future climate change (Kremer *et al*., 2012; Aitken and Bemmels, 2016). Thus, mixing seeds from multiple sources may be an appropriate strategy for increasing diversity and buffering climate change for marginal populations of *P. macroptera* (Aitken and Bemmels, 2016; Martins *et al*., 2018). Meanwhile, donor populations should be carefully selected, so that transplanted individuals are genetically compatible with the new environment predicted in future reconstructive management (Fredriksen *et al*., 2020).

## SUPPLEMENTARY DATA

Supplementary data are available online at https://academic.oup.com/aob and consist of the following.

Figure S1: ADMIXTURE bar plots of the proportion of genetic membership for each ancestry. Figure S2: *F*_ST_ outlier SNPs in *P. macroptera* identified by PCADAPT and OutFLANK. Figure S3: unique and shared outlier SNPs for the top 20 % *F*_ST_ SNPs identified by PCADAPT and OutFLANK. Figure S4: Manhattan plot of SNPs called by BAYENV in *P. macroptera* with eight environment variables. Figure S5: Manhattan plot of SNPs called by LFMM in *P. macroptera* with eight environmental variables. Figure S6: unique and shared outlier SNPs associated with the top four environmental variables. Figure S7: cumulative importance of genetic variation along environmental gradients in *P. macroptera*. Figure S8: partial redundancy analysis by condition geography using all SNPs, *F*_ST_ SNPs identified in PCADAPT and OutFLANK, and GEA SNPs identified in BAYENV and LFMM. Figure S9: prediction of genetic offset to future climate change based on five environment variables for all SNPs and GEA SNPs. Table S1: summary of statistical information on sequencing quality for 28 populations of *P. macroptera.* Table S2: details of population locations, sample size and current environmental variables for 28 populations of *P. macroptera.* Table S3: predicted environmental variables for the years 2081–2100 under two shared socio-economic pathways for *P. macroptera.* Table S4: genetic information statistics on mapping rate and missing rate of 160 individuals in 28 populations. Table S5: statistical information for 8244 SNPs. Table S6: functional annotation of 8244 SNPs based SnpEff software. Table S7: genetic diversity for 28 populations of *P. macroptera*. Table S8: unbiased estimation of nucleotide diversity for NWY, WS and QDT lineages of *P. macroptera*. Table S9: outlier SNPs detected by *F*_ST_-based methods. Table S10: functional descriptions of genes associated with SNPs identified by *F*_ST_- and GEA-based methods. Table S11: outlier SNPs detected by GEA-based methods. Table S12: accuracy importance of each environmental variable identified by GF modelling for *P. macroptera*. Table S13: partial Mantel test in *P. macroptera* conditioned with environmental and geographical distance. Table S14: partitioning of the variance and accumulated constrained eigenvalues associated with environment based on partial redundancy analysis for all SNPs, *F*_ST_ SNPs, GEA SNPs, and all outlier SNPs. Table S15: summary of risk of non-adaptedness calculated for SSP126 and SSP585 in *P. macroptera* populations based on future climate predictions for 2081–2100.

mcad083_suppl_Supplementary_Figures

mcad083_suppl_Supplementary_Tables
