# Supplementary material for: Adaptive divergence and genetic vulnerability of relict species under climate change: a case study of Pterocarya macroptera
Source: Ann Bot. 2023 Jul 6;132(2):241–54. doi: 10.1093/aob/mcad083 (PMC10583204; doi:10.1093/aob/mcad083)

Supplementary Data Fig. S1 ADMIXTURE bar plots for the proportion of genetic membership to each ancestry assuming (K = 2, 3) ancestral populations. (A) the optimal clustering number (K value). (B, C) Each lineage is divided into a different colour to indicate the genomic likelihood belonging to an ancestral *P. macroptera* population.


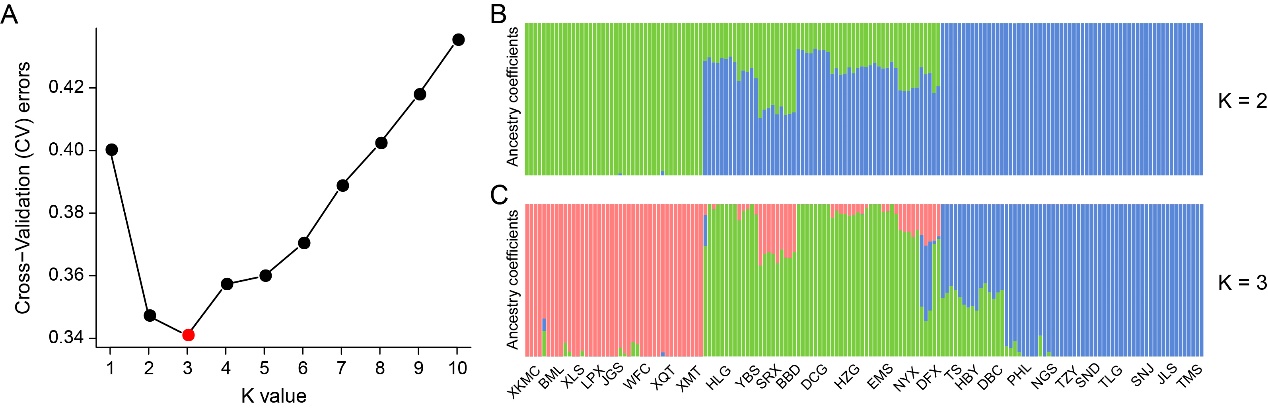


Supplementary Data Fig. S2 *F*_ST_ outlier SNPs in the *P. macroptera* identified by (A) PCADAPT and (B) OutFLANK.


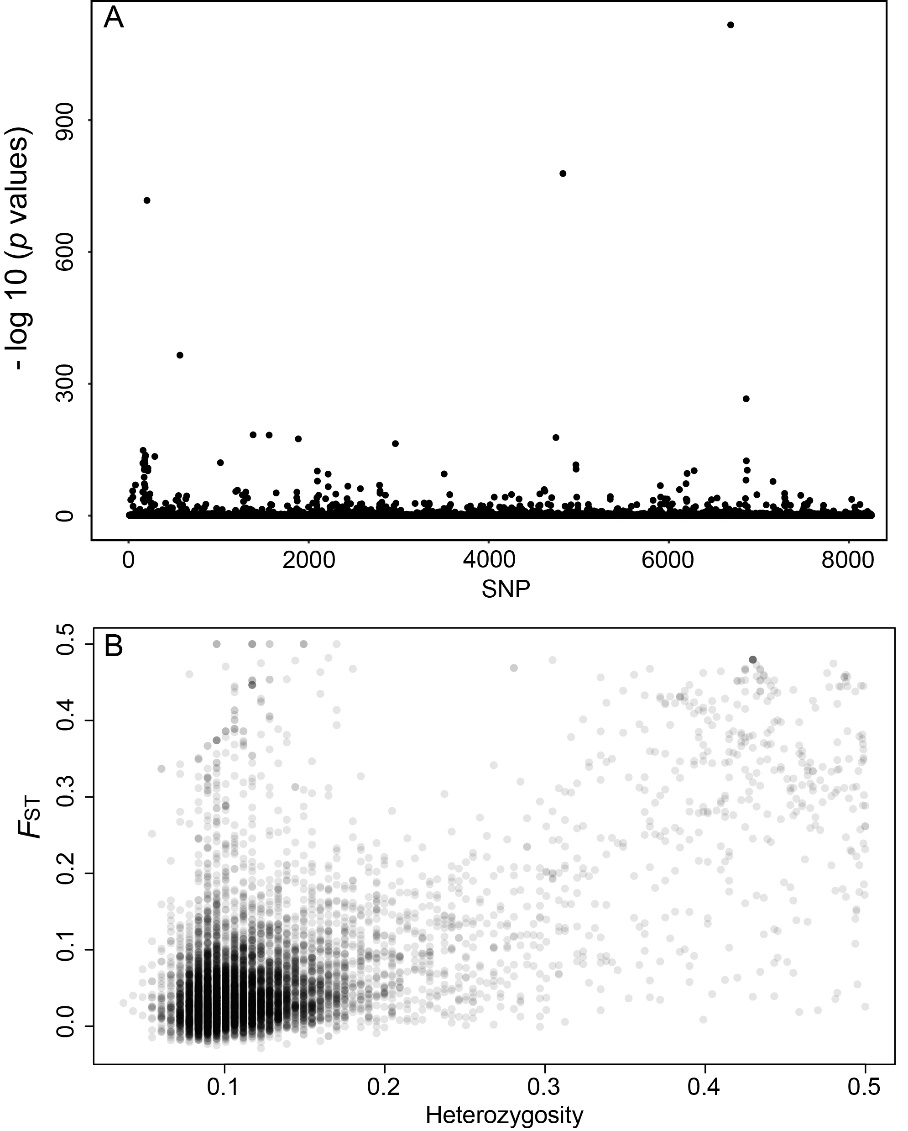


Supplementary Data Fig. S3 Unique and shared outlier SNPs for the top 20% *F*_ST_ SNPs identified by PCADAPT and OutFLANK.


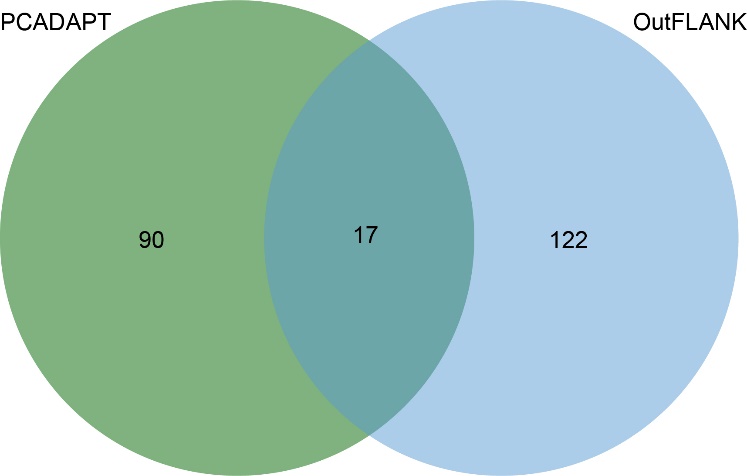


Supplementary Data Fig. S4 Manhattan plot of SNPs called by BAYENV in the *P. macroptera* with eight environment variables. The X-axis represents all SNPs and the y-axis represents the log_10_ (Bayes Factor) for each SNP.


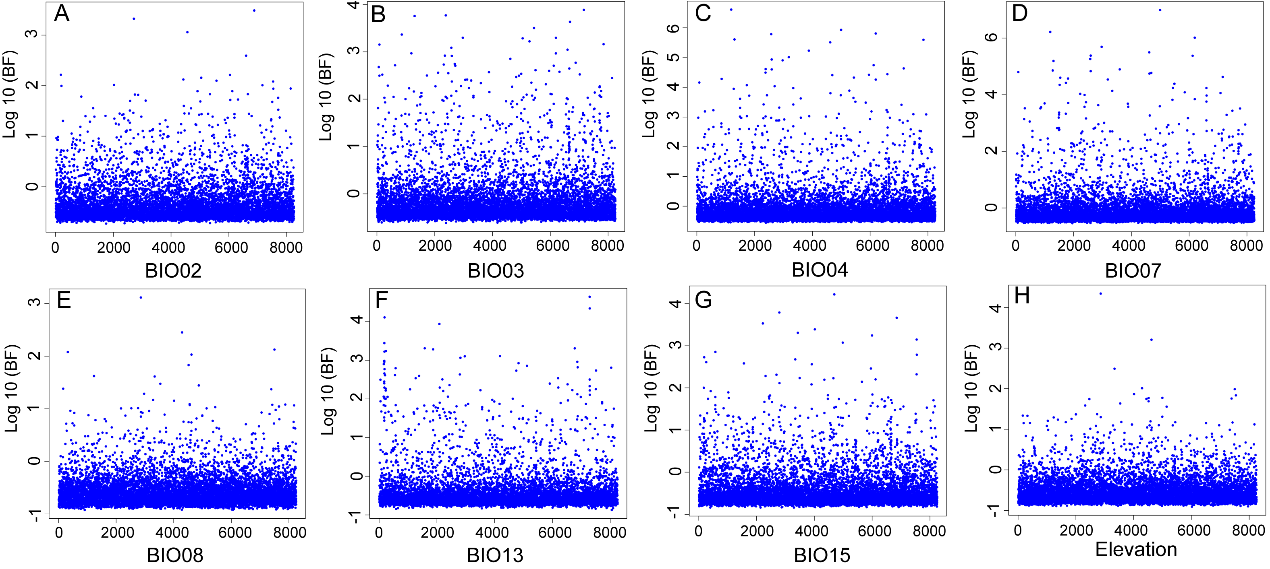


Supplementary Data Fig. S5 Manhattan plot of SNPs called by latent factor mixed modelling (LFMM) in the *P. macroptera* with eight environment variables. The X-axis represents all SNPs and the y-axis represents the -log_10_ *p-value* for each SNP.


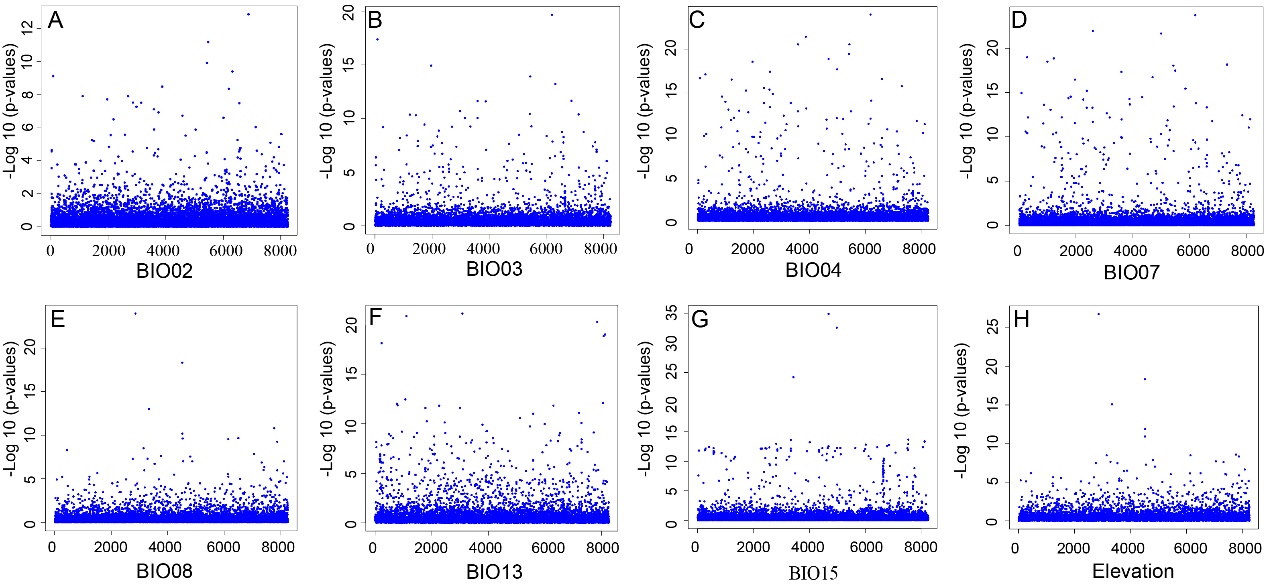


Supplementary Data Fig. S6 Unique and shared outlier SNPs associated with top four environmental variables. Venn diagram showing the SNPs detected by (A) BAYENV, (B) latent factor mixed modelling (LFMM), and (C) SNPs detected in BAYENV and LFMM were associated with all four environmental variables.


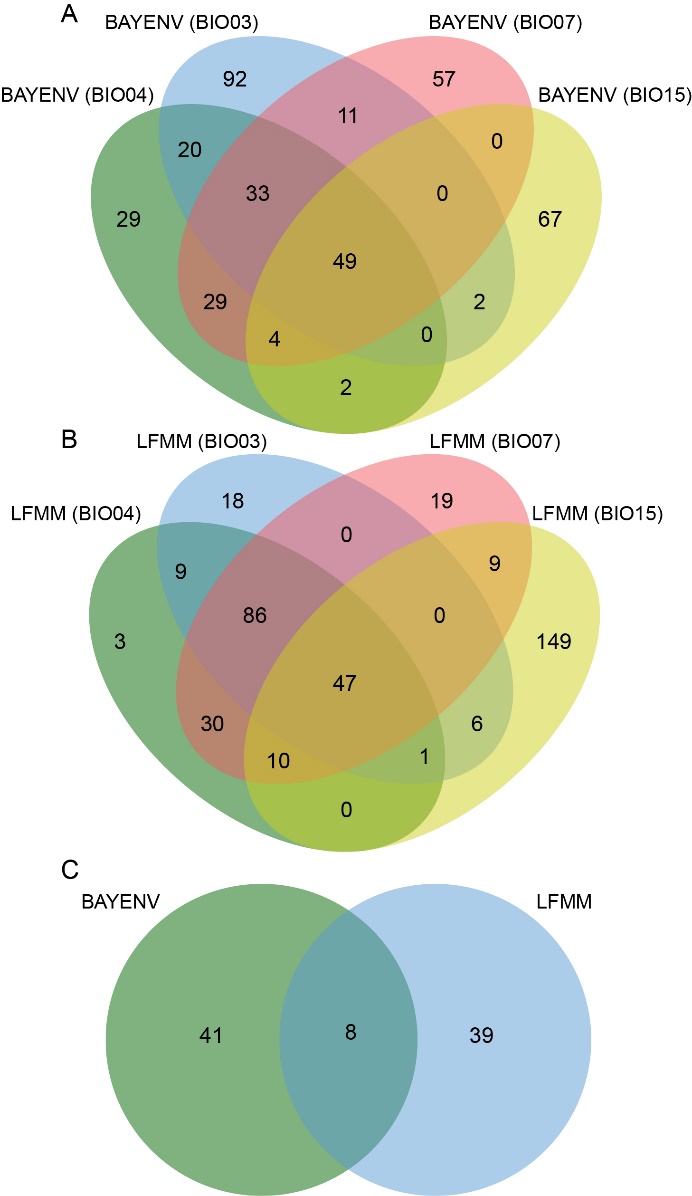


Supplementary Data Fig. S7 Cumulative importance of genetic variation along environmental gradients in *P. macroptera*.


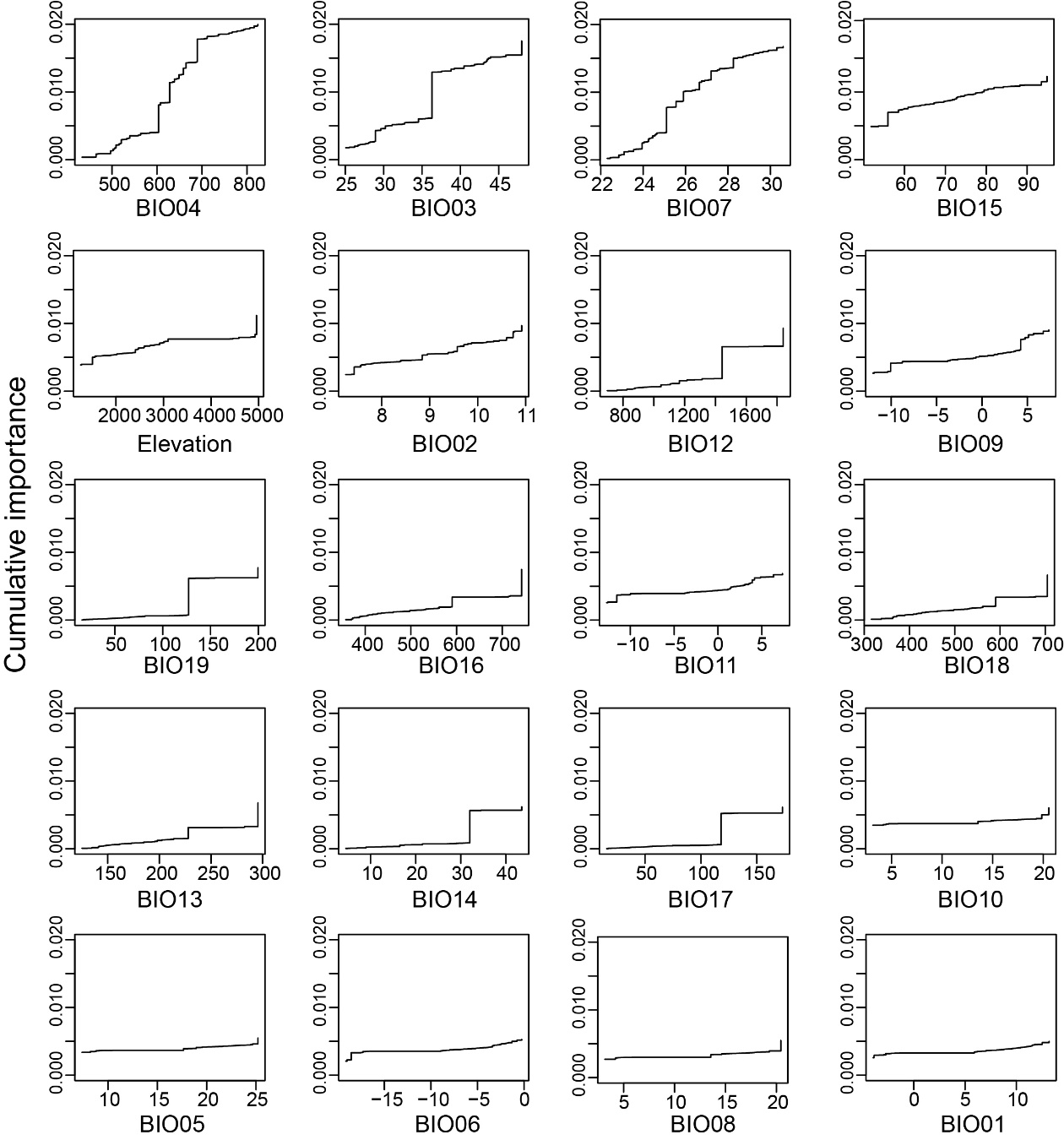


Supplementary Data Fig. S8 Partial redundancy analysis (RDA) by condition geography using (A) all SNPs, (B) *F*_ST_ SNPs identified in PCADAPT and OutFLANK, and (C) GEA SNPs identified in BAYENV and latent factor mixed modelling (LFMM). This plot shows the first and second RDA axes with individuals as coloured circles and the environmental variables as black vectors.


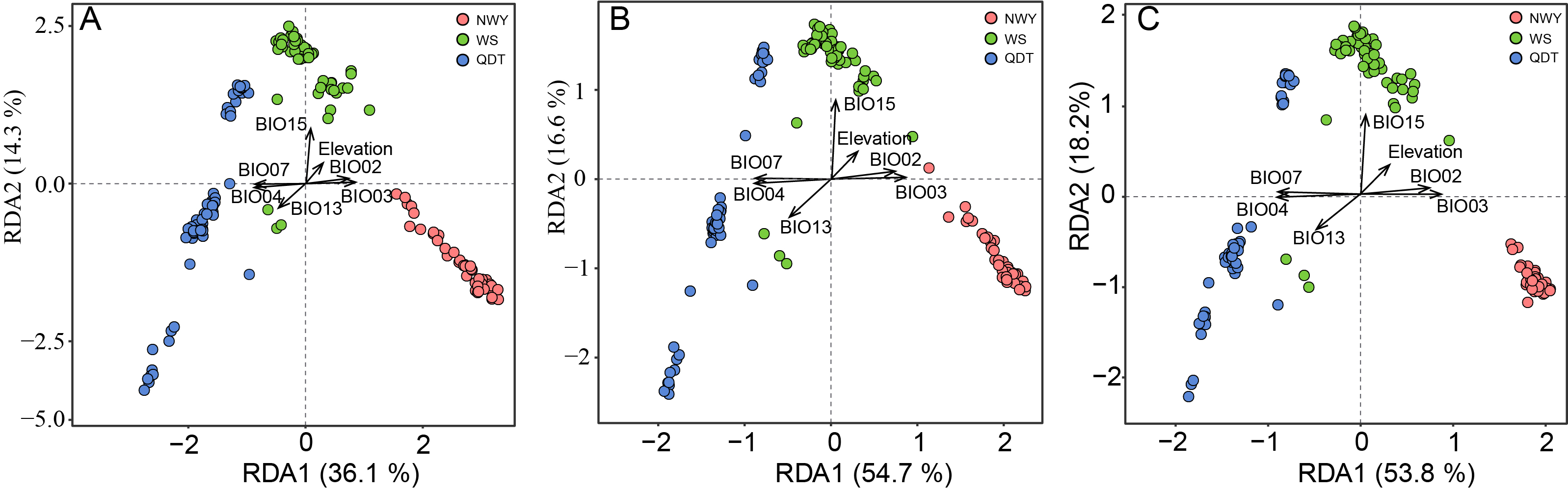


Supplementary Data Fig. S9 Prediction of genetic offset to future climate change based on five environment variables for (A, B) all SNPs and (C, D) GEA SNPs. (A) and (C) reflect scenario shared socioeconomic pathway (SSP)126 2081-2100; (B) and (D) reflect scenario SSP585 2081-2100. Red and blue indicate high and low genomic offset, respectively. Black dots represent sampling sites on the geographic map, with squares, triangles and circles representing Northwest Yunnan (NWY), Western Sichuan (WS) and Qinling-Daba-Tianmu Mountain (QDT) lineage, respectively.


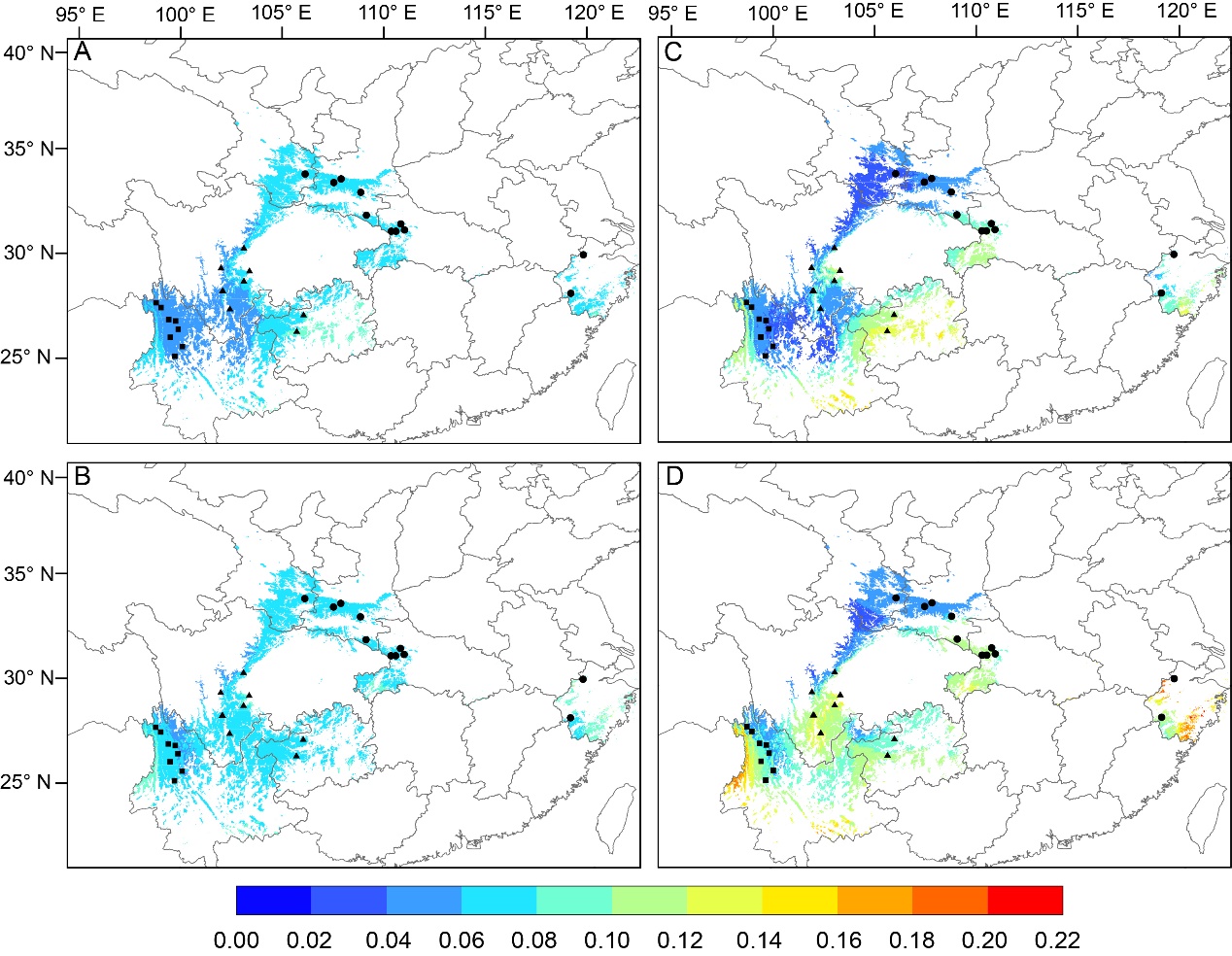

Supplement: mcad083_suppl_Supplementary_Figures [file mcad083_suppl_supplementary_figures.docx]
